# Metabolic follow-up at one year and beyond of women with gestational diabetes treated with insulin and/or oral hypoglycaemic agents: study protocol for the identification of a core outcomes set using a Delphi survey

**DOI:** 10.1186/s13063-018-3059-8

**Published:** 2019-01-05

**Authors:** Delia Bogdanet, Aoife Egan, Narjes Fhelelboom, Linda Biesty, Shakila Thangaratinam, Eugene Dempsey, Caroline Crowther, Declan Devane, Fidelma Dunne

**Affiliations:** 1 College of Medicine, Nursing and Health Sciences, National University of Ireland Galway, Galway, Ireland; 2 Queen Mary, University of London, Women’s Health Research Unit London, London, UK; 3 Paediatrics and Child Health, University College Cork, Cork, Ireland; 4 Liiggins Institute, The University of Auckland, Auckland, New Zealand

**Keywords:** Core outcome set, Gestational diabetes, Insulin, Oral hypoglycaemic agents

## Abstract

**Background:**

Gestational diabetes (GDM) is associated with an increased lifetime risk for the development of glucose abnormalities, metabolic syndrome, cardiovascular disease, depression and tumours. Despite this high risk of additional comorbidities, there is no standardised approach to the long-term follow-up of women with a previous diagnosis of GDM. Also, there is no standardisation of outcome selection and reporting in studies involving this population. This increases the risk of reporting bias and reduces the possibility of meaningful comparisons between studies. The aim of this study is to develop a protocol for a core outcome set (COS) for the metabolic follow-up at 1 year and beyond of women with previous GDM treated with insulin and/or oral hypoglycaemic agents.

**Methods/design:**

This protocol will describe the steps that will be taken in order to develop the COS. The study will consist of three parts: (1) A systematic review of the literature of the outcomes reported in previous randomised controlled trials of the follow-up at 1 year and beyond of women with GDM treated with insulin and/or oral hypoglycaemic agents; (2) A three-round, online Delphi survey with key stakeholders in order to prioritise these outcomes; and (3) A consensus meeting where the final COS will be decided.

**Discussion:**

The proposed protocol is the first step in developing a COS that will bring consistency and uniformity to outcome selection and reporting in GDM women treated with insulin and/or oral hypoglycaemic agents.

**Electronic supplementary material:**

The online version of this article (10.1186/s13063-018-3059-8) contains supplementary material, which is available to authorized users.

## Background

In Europe, the prevalence of gestational diabetes (GDM) is approximately 13% [1, 2]. Medical nutritional therapy is the keystone of treating GDM as it maintains desired glycaemic goals in 80–90% of GDM women [3]. The remaining 10–20% will require insulin and/or oral hypoglycaemic agents.

GDM is associated with a significant lifetime risk of progression to type 2 diabetes. A meta-analysis of cohort studies conducted over the last 40 years showed a relative risk of 7.7 for the future development of type 2 diabetes (T2DM) in women with a history of GDM compared with women with normal glucose tolerance (NGT) in pregnancy [4]. Women with previous GDM are at risk of developing metabolic syndrome [5] and cardiovascular disorder [6] in later life. Some studies show increased postpartum depression among women with GDM [7, 8]. There is also an increasing body of literature associating a previous diagnosis of GDM with the development of tumours, particularly breast and endometrial tumours [9, 10].

Because GDM may represent pre-existing undiagnosed type 2 diabetes, women with GDM should be screened for persistent diabetes or prediabetes at 6–12 weeks postpartum using nonpregnancy criteria and every 1–3 years thereafter depending on risk factors [11]. However, there is no standardised approach to the long-term follow-up of women with a previous diagnosis of GDM. More so, there is no standardisation of outcome selection and reporting in studies involving this population.

**Fig. 1:**
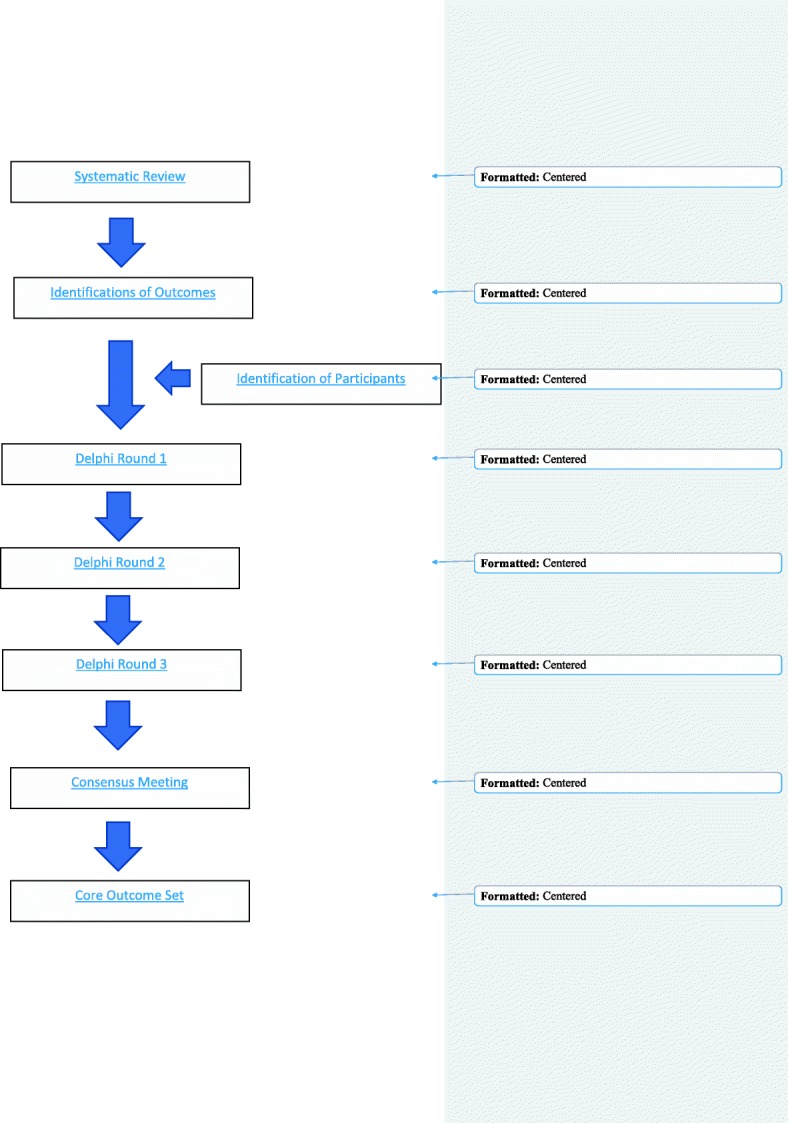
Study process

### Development of a core outcome set

A core outcome set (COS) defines the minimum set of outcomes that should be consistently measured and reported in clinical trials. The existence of a COS does not mean that only these outcomes should be measured. However, a minimum, or core, set of outcomes will provide greater consistency of reporting in clinical trials and more evidence to contribute to meta-analyses. Also, this will reduce the risk of reporting bias by consistently measuring and reporting these outcomes.

The outcomes relevant to post-pregnancy care are numerous and include both maternal and child outcomes. There is significant diversity in the outcomes measured and reported in the studies evaluating long-term follow-up of women with a previous diagnosis of GDM. This heterogeneity in outcomes is highlighted in a Cochrane systematic review evaluating the effects of exercise or exercise and diet on preventing T2DM [12]. Orozco and colleagues noted that of eight studies only six recorded the Body Mass Index (BMI), only four recorded the waist to hip ratio (WHR), five recorded the total cholesterol value and six recorded blood pressures. This variation in outcome selection makes comparison and synthesis of work difficult and amplifies the bias whereby researchers might select outcomes that only answers one research question and report only favourable results [13, 14]. A COS will facilitate the comparison and facilitate the synthesis of appropriate studies. The development of such COS is facilitated and supported by the Core Outcome Measures in Effectiveness Trials Initiative (COMET), which was launched in 2010 and brings together researchers interested in the development, application and promotion of COS while minimising duplication of effort [15]. In 2014, the editors of over 50 journals began the Core Outcomes in Women’s and Newborn Health (CROWN) [16, 17] initiative, the purpose of which is to encourage researchers to develop COSs, report the results of the core outcome sets and facilitate embedding of COS in clinical practice.

Another important argument for the development of COS is that outcomes reported for trials may not reflect the endpoints important for health service users. In previous studies, patients identified outcomes important to them, outcomes that might not have been considered by clinicians [18–20].

### Study aim

The aim of this article is to present a protocol for a study to develop a COS for trials and other studies in the follow-up at 1 year and beyond of women with gestational diabetes treated with insulin and/or oral hypoglycaemic agents. This study focusses only on women with GDM treated with insulin and oral hypoglycaemic agents as this population has more glucose abnormalities and is more likely to progress to T2DM, obesity and metabolic syndrome.

## Methods/design

Ethics: ethical approval to conduct this study was sought and obtained from the Ethics Committee at Galway University Hospitals (home institution of the study’s principal investigator).

### Part 1: Systematic review to generate a list containing possible relevant outcomes

#### Systematic review question

What are the outcomes reported in clinical trials of the follow-up at 1 year and beyond of women with gestational diabetes treated with insulin and/or oral hypoglycaemic agents?

### Methods

Using a broad-based search strategy, the following databases will be searched for relevant studies: the Cochrane Central Register of Controlled Trials (CENTRAL), the Cumulative Index to Nursing and Allied Health Literature (CINAHL), PubMed, Embase and Web of Science. ClinicalTrials.gov will also be searched for relevant, ongoing trials. The advantages conferred by using CENTRAL in addition to the other databases are that trials from other sources of research are hand searched, and controlled trials from these are included. This improves the chances of identifying all relevant studies. Key terms used to guide the search will include ‘gestational diabetes’, ‘GDM’, ‘insulin’, ‘oral hypoglycaemic’, ‘oral antihyperglycaemic’, ‘treatment’, ‘pharmacological’, ‘medication’, ‘antidiabetic’, ‘metformin’ ‘glyburide’,’ outcome’, ‘follow-up’ and ‘postpartum’ combined as appropriate using the Boolean operands ‘AND’ and ‘OR’. There are no date restrictions for study inclusion. An example for the search strategy has been provided in the Additional file 1.

The reference lists of all relevant studies will be searched for additional relevant studies not retrieved from the electronic database search. Language restrictions will not be applied to the search strategy; however, selection of relevant articles will be restricted to English language publications. Searching all languages will enable us to identify the extent of potentially eligible additional studies that will not be included and consider if this presents a source of language bias.

### Types of studies

Including all types of studies will generate an extremely large list and seeing that clinical trials are more likely to have clearly defined outcomes, we will only include randomised control trials (RCTs) and RCT follow-up studies in our systematic review. Studies by any other design other than RCT will be excluded. In line with prior work in this area, we will also exclude review reports and reports of conference proceedings, studies with less than 10 participants or abstracts when their complete description of the trial or study is absent.

### Types of participants

Participants will be women with previously diagnosed gestational diabetes by any internationally agreed criteria (the World Health Organisation (WHO),the International Association of the Diabetes and Pregnancy Study Groups (IADPSG), the National Institute for Health and Care Excellence (NICE), Carpenter and Coustan, the American Diabetes Association (ADA),the American College of Obstetricians and Gynaecologists (ACOG), The National Diabetes Data Group (NDDG), Hyperglycemia and Adverse Pregnancy Outcome (HAPO), O’Sullivan and Mahan) treated with insulin and/or oral hypoglycaemic agents in the index follow-up period. We will not restrict the search to any particular screening or diagnosing criteria for gestational diabetes to increase usefulness of the COS internationally.

### Exclusion criteria

Exclusion criteria will include studies of women with a pre-pregnancy diagnosis of diabetes and studies that only include participants treated with medical nutritional therapy only.

### Study assessment

An initial selection of studies identified in the search will be performed using the predetermined review inclusion criteria. Due to the absence of a study design filter, we expect that a large number of records will be identified by our search strategy. In the first step, all identified study titles will be reviewed and ineligible studies excluded (FPD and DB). In the second step of the review, the remaining studies will be reviewed by two reviewers (FPD and DB) who will independently assess the titles and abstracts of studies included at this stage. Full texts of studies meeting the inclusion criteria, or where there is uncertainty regarding inclusion at title and abstract screening, will be retrieved and reviewed independently by two authors (FPD and DB) with final decisions on inclusion or exclusion achieved through consensus. In cases of disagreement, a third independent reviewer will be consulted (DD).

### Data extraction

Outcomes will be identified within the ‘Methods’ and ‘Results’ sections of each paper and for each outcome we will assess how it was defined and measured, the definition used, the number of participants in which it was measured, the indicators and/or tools used to measure the outcomes, the time points or periods of outcome measurement and whether it was stated in both the methods and results. Additional data that will be extracted includes: study design, author details, year and journal of publication. The individual outcomes will be categorised under broader outcome domains.

Two review authors (FPD and DB) will extract data independently, review the data together, assess consensus and ensure that all outcomes have been identified. Disagreement will be resolved through discussion. Where a resolution is not possible, a third reviewer will be consulted.

### Data analysis

All eligible studies will be tabulated and the identified outcomes presented along with their definitions. We wish to establish whether outcomes are defined and, if they are, whether there is a difference in defining terms between studies which would make comparison of these outcomes between trials more difficult.

All outcomes identified in the studies will be included. These outcomes will be categorised under appropriate outcome domains which will be decided upon by the authors. Within each domain, we will be able to evaluate both how many different outcomes have been used to reflect that domain and the frequency of selection for each individual outcome, and the times at which they were measured will be documented. The outcome domains will be reviewed by the Study Advisory Group (SAG) to assess suitability of the domain name and grouping of outcomes. The SAG will include representatives from researchers, healthcare professionals and patients.

### Part 2: Developing a consensus on outcomes important to different stakeholders using a Delphi survey and consensus meeting

#### Delphi survey

We will use a Delphi survey to develop consensus on the most important outcomes from those identified in the systematic review [21]. The Delphi survey was developed originally by the RAND Corporation in the 1950s in order to forecast the influence of technology on warfare [22]. It is an iterative consensus technique, which comprises successive questionnaires answered by a panel of participants with relevant expertise. Questioning takes place in rounds, and after each round of questions, an anonymous summary of the responses is fed back to the group. Individual participants may then decide to keep their original answers or to change their opinion in the subsequent round. Participants will not be able to identify other participants or individual responses.

This technique has the advantage over a roundtable meeting in that it can avoid the situation where certain individuals can dominate a discussion or where other individuals feel obliged to agree with the opinions of more senior members. It also facilitates wide international participation and increased numbers of stakeholders informing the prioritisation process. To improve efficiency, the questionnaire will be completed online using an appropriate software for online survey design.

The list of potential outcomes finalised in part 1 will be formatted into a list of outcomes with a response designed to allow the participants to rate each one of the outcomes on a 9-point Likert-type scale, with higher values representing increased importance for inclusion of the outcome in the COS. Following review of the outcome list by the research group (DB, AE, NF, LB, CC, DD and FPD), the list will be circulated to the SAG. The SAG, composed of key stakeholders, will be asked to comment on the overall list of outcomes and the suitability of the domains under which they are grouped. They will additionally be asked to list any other outcomes that they think should be included. These other outcomes will be added to the list for inclusion in the Delphi survey. Members of the SAG will not participate in the Delphi exercise, as they had a role in study design, which may influence scoring. Instead, they will be invited to participate in the final consensus meeting.

### Participants

The panel of participants includes:

- Women (service users); they will have had a diagnosis of gestational diabetes and will have been treated with insulin and/or oral hypoglycaemic agents for this diagnosis. Recruitment will include listed groups of diabetes services users (e.g. Diabetes Ireland) accessed via the electronic discussion email list manager. The manager will be emailed information on the survey with a request to distribute an invitation email to members on their email lists. The list managers will have an opportunity to contact the researcher directly to clarify any issues or to seek further information about the survey and the research before deciding. The distribution of the survey will be at the discretion of the email list manager. Clinicians will also be asked to invite service users to participate via their pregnancy and diabetes care clinical teams
- Clinicians will include endocrinologists, obstetricians, paediatricians, neonatologists, general practitioners and practice nurses, diabetic nurses, midwives, dieticians and physiotherapists
- Researchers with expertise in diabetes care from DPSG (Diabetes in Pregnancy Study Group), IADPSG, FIGO (International Federation of Gynaecology and Obstetrics) and EBCOG (European Board and College of Obstetrics and Gynaecology) will be invited to participate
- Policy-makers from the HSE (Healthcare Service Executive), IDF (International Diabetes Federation) and WHO (World Health Organisation) will also be invited to participate

Clinicians and researchers will be invited from national and international specialist centres. Clinical leads will be identified and recruited through the DPSG, which is a study group of the European Association for the Study of Diabetes (EASD), IADPSG, FIGO and EBCOG. Clinicians will also be identified through journal email lists, national training bodies and national diabetes societies. Clinical leads will be invited to participate by email and will be asked to forward the study details to other members of their pregnancy care teams.

Participants from all the above groups will be asked to forward the invitation to others whom they regard as having the required expertise. Although there is currently no standard method for sample size calculation in the Delphi process, we will aim for a total of 180 participants, including at least 30 service users. Efforts will be taken to maximise the response rate across stakeholder groups.

Each participant will be emailed an invitation outlining the study, its importance and a link to the online survey. Informed consent to participate in the study will be obtained from each participant when registering for the online Delphi questionnaire. The consent process will take place before any answer is submitted. We will underline the importance of completing the whole Delphi survey process, and generic reminder emails will be sent to aid completion of each round. A unique identifier will be assigned to each participant, tracked to their email address, which will allow monitoring of attrition at each round.

### Delphi study round 1

The online questionnaire will request the participant’s name, email address and centre with to which they are aligned. Participants will be asked to identify the stakeholder group and subgroup to which they belong. They will be asked to complete the questionnaire within 3 weeks. A reminder email will be sent the end of week 2 to prompt completion of the survey.

The questionnaires will contain lay terminology alongside clinical terms to assist women in understanding complex terminology. The list of outcomes to be scored will be ordered alphabetically to avoid weighting of outcomes caused by the order in which they are displayed. There will be an option for a participant to add up to two additional outcomes not already included together with a score for each outcome added.

Participants will be asked to score each of the outcomes listed using the Grading of Recommendations Assessment, Development and Evaluation (GRADE) scale which involves a 9-point scale to rank importance. Scores will be grouped into 1–3 = limited importance, 4–6 = important but not critical or 7–9 = critical.

### Analysis of round 1

The results of round 1 will be summarised using descriptive statistics, including the proportion of participants from each stakeholder group scoring for each rating point on the Likert scale (i.e. for each point from 1 to 9). The additional outcomes listed will be reviewed by the researchers (FPD and DB). If they have been proposed by two or more participants and if they are deemed to represent a new outcome based on this review, they will be included for round 2 and the SAG will be consulted to review if appropriate. All outcomes will be carried forward to the second round with first round scores displayed for each outcome and the distribution of scores from each stakeholder group. Continuation to round 2 will be considered based on the response to round 1. If a low number of responders (< 10) is observed for one or more stakeholder groups, the Delphi protocol for future rounds will be reviewed and revised. If there is only one stakeholder group with a small number of respondents, then that group may be classified together with another stakeholder group following consultation with the SAG to ensure appropriateness of grouping.

### Delphi study round 2

Participants who respond to round 1 will be forwarded the round 2 questionnaire and asked to complete it online. Each participant will be presented with the number of respondents and distribution of scores for each outcome per stakeholder group. They will also be shown their score from round 1, asked to consider responses from the other members of the stakeholder groups and invited to re-score the outcome.

### Analysis of round 2

The total number of participants invited to take part in round 2 will be recorded. The results of round 2 will be summarised using descriptive statistics. For each outcome, the number of participants who have scored the outcome and the distribution of scores will be noted. Consensus to carry an outcome through to the COS will be defined as more than 70% of participants scoring its importance as 7–9 and less than 15% scoring it as 1–3.

### Delphi study round 3

Participants who respond to rounds 1 and 2 will be forwarded the round-3 questionnaire and asked to complete it online. Again, each participant will be presented with the number of respondents and distribution of scores for each outcome for each stakeholder groups. They will also be shown their score from round 2, asked to consider responses of other members of the stakeholder group and asked to re-score the outcomes.

### Analysis of round 3

The total number of participants taking part will be recorded. The results of round 3 will be summarised using descriptive statistics. For each outcome, the number of participants who have scored the outcome and the distribution of scores from each stakeholder group will be noted. Consensus to carry an outcome through to the COS will be defined as more than 70% of participants scoring its importance as 7–9 and less than 15% scoring it as 1–3. Each outcome will be classified as ‘consensus in’, ‘consensus out’ or ‘no consensus’. Although these cut-offs are subjective, they have previously been described in a COS protocol [23] and are prespecified here.

### Consensus group meeting

#### Methods

This final phase will involve a face-to-face meeting with key stakeholders. The meeting participants will be selected to ensure that all stakeholder groups are represented reasonably. The participants will be invited from among those who completed all rounds of the Delphi study. The results obtained from each round of the Delphi survey will be presented. The objective of the consensus meeting is to discuss outcomes on which there were incongruous opinions in round 3 of the Delphi study and to agree on a list of final outcomes which will constitute the COS. Discussion of each reporting item will be followed by an anonymous scoring method by those at the consensus meeting. Similar to the Delphi stage, items will be considered as ‘consensus in’ if 70% of the consensus meeting participants vote in favour of the item to be included in the COS. The meeting will also agree on publication and dissemination strategies.

### Participants

The consensus group will include 10–20 participants and members of the SAG. A half-day meeting is planned to achieve effective consensus. The facilitator will ensure that the meeting is collaborative, egalitarian, comprehensive and participatory. A flow diagram of the study methods has been provided in Fig 1.

## Discussion

There is currently no COS for studies assessing the follow-up of women diagnosed with gestational diabetes treated with insulin/oral hypoglycaemic agents. The aim of development of such a COS in this clinical area is to improve the interpretation and comparison of future studies while reducing the risk of outcome reporting bias and heterogeneity between studies. We will involve key stakeholders and use recognised techniques to ensure that the resulting COS is suitable and accepted in future research studies.

### Study status

The study plan is to complete the systematic review by December 2017 and development of the COS by October 2018.

## Additional file

Additional file 1: Search strategy examples. (DOCX 14 kb)

## Abbreviations

ACOG: American College of Obstetricians and Gynaecologists
ADA: American Diabetes Association
CENTRAL: Cochrane Central Register of Controlled Trials
CINAHL: Cumulative Index to Nursing and Allied Health Literature
COMET: Core Outcome Measures in Effectiveness Trials Initiative
COS: Core outcome set
CROWN: Core Outcomes in Women’s and Newborn Health
DPSG: Diabetes in Pregnancy Study Group
EASD: European Association for the Study of Diabetes
EBCOG: European Board and College of Obstetrics and Gynaecology
FIGO: International Federation of Gynaecology and Obstetrics
GDM: Gestational diabetes
GRADE: Grading of Recommendations Assessment, Development and Evaluation
HAPO: Hyperglycemia and Adverse Pregnancy Outcome
HSE: Health Service Executive
IADPSG: International Association of Diabetes in Pregnancy Study Group
IDF: International Diabetes Federation
NDDG: The National Diabetes Data Group
NGT: Normal glucose tolerance
NICE: National Institute for Health and Care Excellence
RCT: Randomised controlled trial
SAG: Study Advisory Group
T2DM: Type 2 diabetes
WHO: World Health Organisation
WHR: Waist to hip ratio
