# Supplementary material for: Metabolic follow-up at one year and beyond of women with gestational diabetes treated with insulin and/or oral hypoglycaemic agents: study protocol for the identification of a core outcomes set using a Delphi survey
Source: Trials. 2019 Jan 5;20:9. doi: 10.1186/s13063-018-3059-8 (PMC6321696; doi:10.1186/s13063-018-3059-8)
Supplement: Supplementary file 1 — Search strategy examples. (DOCX 14 kb) [file 13063_2018_3059_MOESM1_ESM.docx]

**Search Strategy Examples**

**Pubmed**

1. Gestational diabetes
2. Gestational diabetes OR GDM OR diabetes in pregnancy
3. Insulin
4. 2 AND 3
5. 4 AND follow-up
6. 4 AND outcome
7. 4 AND outcomes
8. 4 AND follow
9. 5 OR 6 OR 7 OR 8
10. 4 AND postpartum
11. 4 AND post partum
12. 4 AND post-partum
13. 9 OR 10 OR 11 OR 12
14. 2 AND treatment
15. 2 AND follow-up
16. 2 AND Oral
17. 2 AND Oral hypoglycemic
18. 2 AND Oral hypoglycaemic
19. 2 AND Oral antihyperglycemic
20. 2 AND Oral antihypergycaemic
21. 2 AND metformin
22. 2 AND medication
23. 2 AND glyburide
24. 2 AND pharmacological
25. 2 AND antidiabetic
26. 2 AND anti-diabetic
27. 2 AND anti diabetic
28. 2 AND acarbose
29. 4 OR 14 OR 16 OR 17 OR 18 OR 19 OR 20 OR 21 OR 22 OR 23 OR 24 OR 25 OR 26 OR 27 OR 28
30. 29 AND follow
31. 29 AND follow-up
32. 29 AND postpartum
33. 29 AND post-partum
34. 29 AND post partum
35. 29 AND future
36. 29 AND risk
37. 35 OR 36
38. 32 OR 33 OR 34
39. 30 OR 31
40. 37 OR 38 OR 39

(plus additional filters provided by the website on the type of study)

**Embase**

((gestational diabetes) OR GDM OR (diabetes pregnancy)) AND (insulin OR metfORmin OR (ORal medication) OR glibenclamide OR (antidiabetic agent) OR treatment OR glyburide OR (ORal hypoglycemic) OR (ORal antihyperglycemic) OR medication OR acarbose OR pharmacological ) AND (follow-up OR outcome OR outcomes OR post-partum OR postpartum OR risk)

(plus additional filters provided by the website on the type of study)
